# Case Report: Positive Outcome of a Suspected Drug-Associated (Immune Mediated) Reaction in a 4-Year-Old Male French Bulldog

**DOI:** 10.3389/fvets.2021.728901

**Published:** 2021-08-20

**Authors:** Line-Alice Lecru, Daniel Combarros, Eloy Castilla-Castaño, Maxence Delverdier, Marie-Christine Cadiergues, Charline Pressanti

**Affiliations:** ^1^Small Animal Clinic, Université de Toulouse, ENVT, Toulouse, France; ^2^INFINITY, Université de Toulouse, CNRS, INSERM, UPS, ENVT, Toulouse, France; ^3^Basic Sciences Department, Université de Toulouse, ENVT, Toulouse, France; ^4^IHAP, Université de Toulouse, INRAE, ENVT, Toulouse, France

**Keywords:** beta-lactams antibiotic, dog, drug reaction, skin, toxic epidermal necrolysis

## Abstract

Toxic epidermal necrolysis (TEN) is a rare and severe life-threatening syndrome characterized by apoptosis of keratinocytes resulting in devitalization of the epidermis affecting more than 30% of skin surface. In humans and animals, this condition is mostly triggered by drugs. Identification of the putative agent and its withdrawal are crucial to successful management of a patient with TEN. In this case study, we report the clinical features, histopathological findings and management of a dog with TEN. A 4-year-old intact male French bulldog presented with acute onset of severe lethargy and cutaneous ulcerations on the footpads, scrotum, and hind limbs associated with marked pain. A Stevens-Johnson syndrome/TEN was suspected and drugs, especially beta-lactams, were withdrawn. Histopathology confirmed the diagnosis of epidermal necrosis. Advanced supportive therapy, pain management and skin care led to rapid remission. Early identification and removal of the suspected medication was crucial to improving TEN prognosis in this dog. Antibiotics (penicillin, ampicillin, cephalexin, and sulfonamides) are frequently involved in adverse cutaneous reactions in dogs. Ideal treatment remains elusive is humans and dogs and this disease has a poor prognosis. Supportive care combined with pain management and treatment of the cutaneous ulcerations is essential.

## Introduction

Toxic epidermal necrolysis (TEN) is a rare and severe life-threatening syndrome characterized by apoptosis of keratinocytes of all epidermal layers resulting in devitalization of the epidermis in more than 30% of the skin surface (1). After a phase of fever and malaise, TEN ultimately results in extensive skin involvement with erythema, necrosis, and detachment of the epidermis and mucosa. The individuals are then at high risk of developing sepsis and toxic shock (2, 3). In humans and animals, TEN, and Stevens Johnson syndrome (SJS) are mostly triggered by drugs (4, 5) and differ from the surface of the epidermal detachement. Identification of the putative agent and its withdrawal are crucial to successful management of a patient with SJS and TEN in particular (6, 7). We aim to report clinical features, histopathological findings and positive outcome of a suspected drug-associated (immune mediated) reaction in a 4-year-old male French bulldog with a dramatic initial presentation.

## Case Description

A 4-year-old intact male French bulldog presented with a 1-week history of lethargy, dysorexia and painful erythematous macules and patches, the epidermis on the scrotum, perianal region, footpads, and hind limbs being readily detachable. One week earlier, a first veterinarian had treated the dog for superficial injuries (erosions, crusts and traumatic linear ulcers) secondary to a road traffic accident with three consecutive subcutaneous injections of amoxicillin [15 mg/kg (15–20 mg/kg)—Clamoxyl suspension®, Zoetis, Malakoff, France] and one subcutaneous injection of steroidal anti-inflammatory [dexamethasone, 0.1 mg/kg (0.1–0.2 mg/kg)—Dexadreson®, Intervet, Beaucouze, France]. A second veterinarian was consulted when new cutaneous lesions (multiple erosive, crusted and circular between 5 and 10 mm in diameter) appeared spontaneously on the ventral abdomen a week later. The dog was then treated with allopurinol [400 mg once daily, (10 mg/kg/twice a day)—Allopurinol Teva 200 mg, TEVA, Paris, France] after a presumptive diagnosis of leishmaniosis. A complete blood count (CBC) and serum biochemistry profile was obtained; the only abnormality identified was a moderate increase of alanine aminotransferase (ALT). The dog also received cephalexin [22.4 mg/kg twice daily (20–30 mg/kg)—Rilexine® 300 mg, Virbac, Carros, France], and a single intramuscular injection of furosemide [4 mg/kg (2.5–5 mg/kg)—Dimazon®, Intervet, Beaucouze, France]. Within a few days the dog's condition was even worse. Severe systemic signs, lethargy, anorexia, weight loss and pain were apparent and the cutaneous lesions rapidly developed into widespread ulcerations. Further subcutaneous injections of amoxicillin [15 mg/kg (15–20 mg/kg)—Clamoxyl suspension®, Zoetis, Malakoff, France] and dexamethasone [0.1 mg/kg (0.1–0.2 mg/kg)—Dexadreson®, Intervet, Beaucouze, France] did not improve the lesions or the general condition, and the dog was referred. He was presented with lethargy, respiratory distress, hyperthermia, and a 4-day history of anorexia.

**Figure 1:**
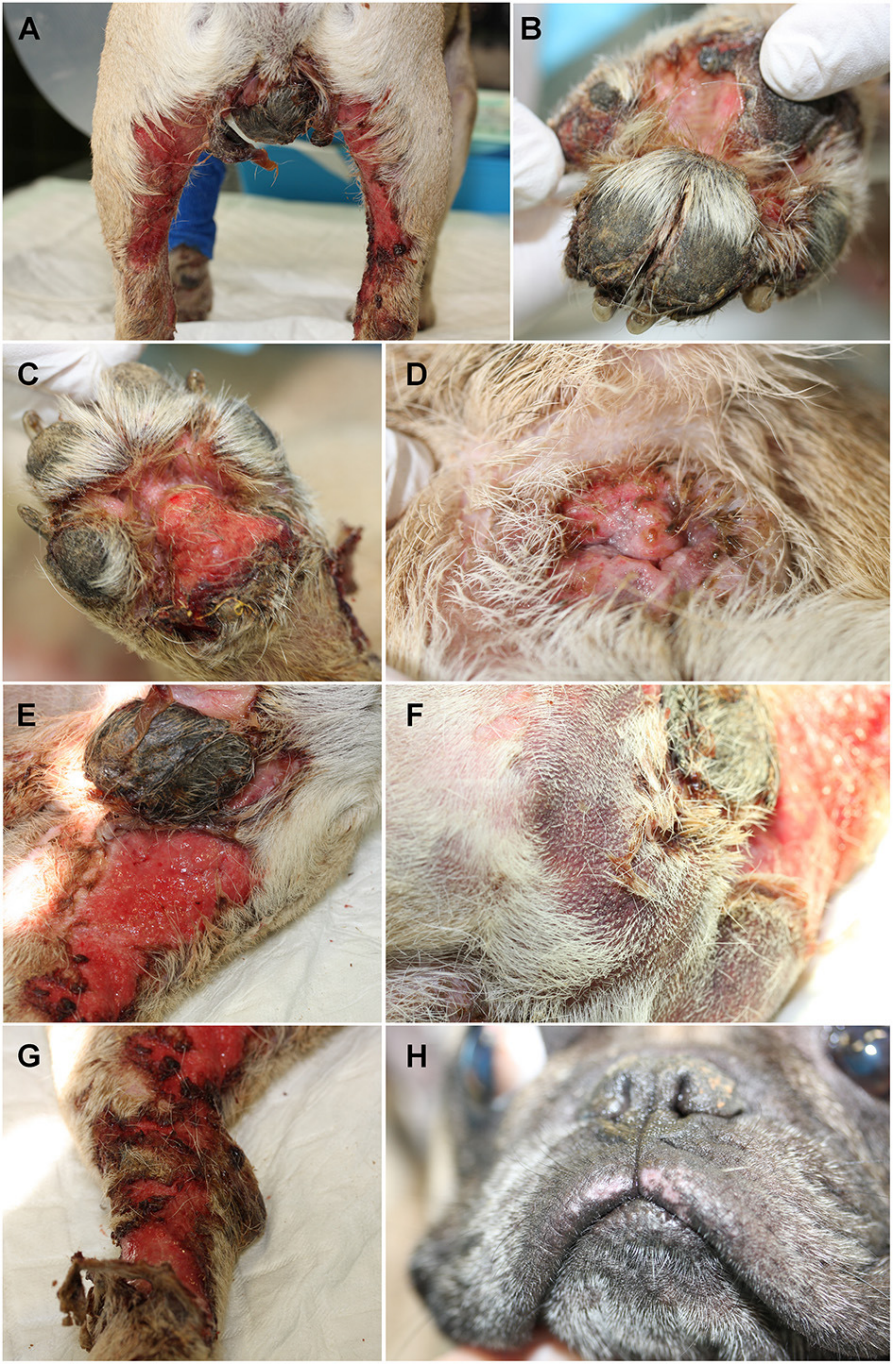
Initial physical examination of a 4-year-old intact male French bulldog. Severe ulcerations involving the inner aspect of the hind legs **(A)** and ulcerations of the interdigital region **(B)** and the footpads **(C)**. Ulcerations, erosions and depigmentation of the perianal region **(D)** and thick crusty material covering the scrotum **(E)**. Purpuric macules and patches were present on the caudal aspects of the thighs **(F)**. Epidermis was sloughing off around the ulcerative lesions **(G)**. Slight depigmentation was visible on the upper lip **(H)**.

On admission at the veterinary teaching hospital, general examination revealed a poor body condition score (2/9–8.6 kg), reluctance to walk, dyspnea and tachypnea, fever (39.8°C) and enlargement of the prescapular lymph nodes. Severe extensive ulcerations were present bilaterally on the inner aspect of the hind legs (Figure 1A), between the digits (Figure 1B) and footpads (Figure 1C). The perianal region was eroded and depigmented (Figure 1D). A thick, adherent crusty plaque covered the scrotum which was almost completely ulcerated (Figure 1E). Large poorly-defined purpuric macules and patches were noted on the caudal aspect of the thigh (Figure 1F). The examination of these lesions was difficult because of the pain; the dog was moving and could not support the palpation. The pruritus was important and estimated 9/10 on a pruritus Visual Analog Scale by the owner. Major epidermal detachment (Figure 1G), very painful necrotizing lesions, and a pseudo-Nikolsky sign epidermal detachment obtained by gentle pressure to the erythematous skin, were apparent together with slight mucocutaneous depigmentation (Figure 1H). The authors estimated a surface of ~1,500 cm^2^ of extended lesions for a total body surface area (BSA) of 0.424 m^2^ (dog's weight: 8.6). Hence, more than 35% of the BSA displayed ulcerations and epidermal sloughing.

**Figure 2:**
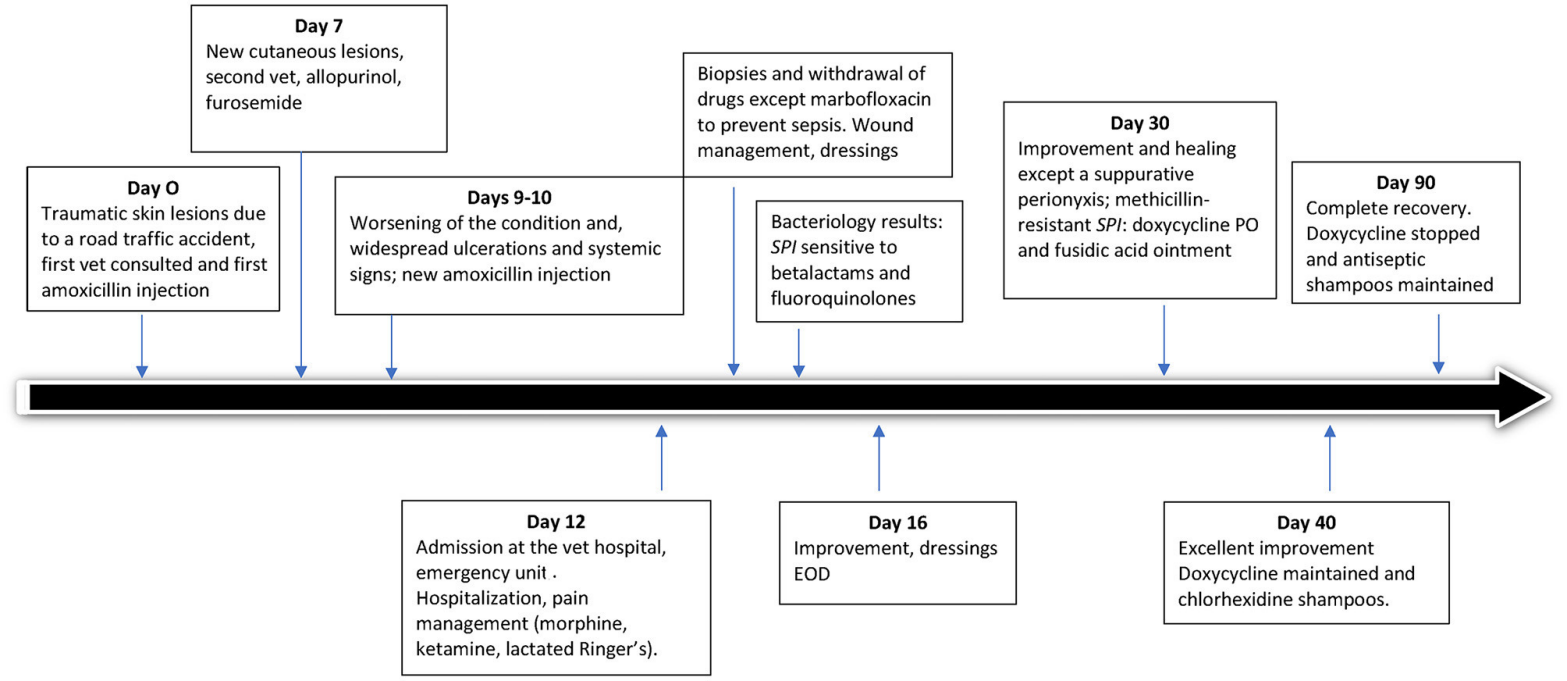
Schematic diagram describing timeline clinical, diagnostic and treatments features of suspected drug-associated (immune mediated) reaction in a 4-year-old male French bulldog. *SPI, Staphylococcus pseudintermedius*.

The timeline of the case is shown in Figure 2.

The differential diagnosis included traumatic burns but was not supported by the history. Toxic epidermal necrolysis (TEN), erythema multiforme (EM) and vasculitis with ischemic necrosis were possible considering the acute onset and widespread nature of the ulcerations. Bacterial or viral infection, staphylococcal toxic shock syndrome and bullous auto-immune diseases (pemphigus and pemphigoid diseases, epidermolysis bullosa acquisita) could be included because of the erosive and ulcerative aspect of the lesions, the mucosal involvement and the systemic illness. Systemic lupus erythematosus could also be considered.

Cytological examination of impression smears from the limb ulcerations showed many neutrophils, eosinophils and intracytoplasmic coccoid bacteria (phagocytosis). A sample was obtained by cotton swab for bacterial culture. Strains of *Staphylococcus pseudintermedius* sensitive to all the antibiotics tested, including betalactams and fluoroquinolones, were isolated. CBC revealed severe neutrophilic leukocytosis (16.10^9^/L). Serum biochemistry was unremarkable except for a huge increase of fibrinogen [8.87 g/L (1.3–4.8)], moderate increase of alkaline phosphatases [346 IU/L (23–212)] and minor hypochloremia [103 mmol/L (110–118)]. Prothrombin time and activated partial thromboplastin time were within the normal ranges. Urinalysis highlighted numerous struvite crystals without proteinuria (urine protein to creatinine ratio = 0.4). Abdominal ultrasonography and thoracic radiographs did not reveal any abnormality. An indirect immunofluorescence test [Enzyme-linked immunosorbent assay (ELISA)] for leishmaniasis was negative. A canine lipase specific SNAP test (ELISA) was normal. Fine-needle aspiration of the enlarged lymph nodes revealed granulomatous adenitis associated with plasma cell hyperplasia.

Cutaneous biopsies were performed on the caudal part of the right forelimb and back thigh under general anesthesia using acepromazine, propofol and isoflurane. Four 6-mm biopsy punch samples were taken from the margins of ulcerative lesions and from the normal skin surrounding the lesions and stained with hemalun and eosin following standard procedures.

A cutaneous drug reaction was highly suspected. A specific investigation of all xenobiotics the dog was receiving at the time of the reaction was made with the owner. Beta-lactams were the single current therapy. Using the Naranjo Algorithm Adverse Drug Reaction Probability Scale (8), a score of 4 of 13 was obtained (Table 1). The causality was considered possible. Beta-lactams were withdrawn.

**Table 1:** Naranjo adverse drug reaction probability scale applied to the case study.

**Question**	**Yes**	**No**	**Unknown**	**Score**	**Reasoning**
1. Are there previous conclusive reports on this reaction?	+1	0	0	**1**	Penicillins as a trigger of TEN have been reported in veterinary and human medicines [veterinary medicine : (9, 10); human medicine : (11–13)]
2. Did the adverse event appear after the suspected drug was administered?	+2	−1	0	**2**	Yes (Timeline schematic)
3. Did the adverse event improve when the drug was discontinued or a specific antagonist was administered?	+1	0	0	**1**	Improvement after discontinuation and supportive care.
4. Did the adverse event reappear when the drug was readministered?	+2	−1	0	**0**	The drug was not readministered.
5. Are there alternative causes that could on their own have caused the reaction?	−1	+2	0	**-1**	Allopurinol as a cause of TEN is described in human medicine (14, 15)
6. Did the reaction reappear when a placebo was given?	−1	+1	0	**0**	No placebo was given.
7. Was the drug detected in blood or other fluids in concentrations known to be toxic?	+1	0	0	**0**	No drug measurements.
8. Was the reaction more severe when the dose was increased or less severe when the dose was decreased?	+1	0	0	**0**	No change in doses.
9. Did the patient have a similar reaction to the same or similar drugs in any previous exposure?	+1	0	0	**0**	No similar reaction previously.
10. Was the adverse event confirmed by any objective evidence?	+1	0	0	**1**	Histopathology.
**Total Score**				**+4**	**Possible association**

Histological examination revealed severe extensive multifocal epidermal necrosis with multifocal epidermal detachment and necrotic material within flaccid vesicles (Supplementary Figure 1A). Some extension of ulcerations to the hair follicles was noted (Supplementary Figure 1B). Minimal dermal inflammation was present. Slight heterogeneous perivascular dermatitis with lymphocytes, neutrophils and plasma cells was observed (Supplementary Figure 1C). Some mild multifocal septal panniculitis was present (Supplementary Figure 1D). Taking together the histological findings and the clinical appearance, the hypothesis of TEN was confirmed.

While awaiting the bacterial culture results, an initial intravenous treatment of marbofloxacin (Marbocyl®, Vetoquinol, Lure, France) at 4 mg/kg (2–4 mg/kg) was administered once daily to prevent sepsis. Still under general anesthesia, ulcer wound management was initiated which consisted of gentle shaving, delicate removal of detached epidermis and disinfection with concentrated chlorhexidine shampoo (Hibiscrub®, Regent medical overseas limited, Manchester, United Kingdom). Soft-adherent dressings with poly-absorbent fibers, UrgoClean® (Elevate SAS, Saint-Romain-en-Viennois, France), which trapped the sloughed residues and absorbed any exudate, and UrgoTul Absorb® (Elevate SAS, Saint-Romain-en-Viennois, France), which stimulated fibroblast proliferation and ensured non-adherence, were changed every day. A high-protein diet was introduced (Royal Canin Obesity ND, Royal Canin, Aimargues, France) to support healing.

By day two, an improvement was already apparent, the skin had started healing and granulation tissue was observed (Supplementary Figure 2). The dog had a good appetite and no digestive disorder or pain was noted. Dressings were changed under sedation every other day for 1 week. Expansion of the cutaneous lesions was halted and analgesics were gradually decreased. Marbofloxacin was maintained based on the sensitivity results received on day 15, 3 days after presentation (Figure 2).

After 2 weeks, the footpads and digits had healed. Localized perionyxis (Supplementary Figure 3A), with dark crusts and suppurative oozing, was noted. Cytological examination of a cotton swab of this lesion showed numerous neutrophils, and intracytoplasmic coccoid bacteria. Bacterial culture revealed a strain of methicillin-resistant *S. pseudintermedius*, only sensitive to fusidic acid, tetracyclines and chloramphenicol. The antibiotherapy was modified and doxycycline (Doxyval®, CEVA, Libourne, France) was administered orally once daily at the recommended dose 10 mg/kg. In the absence of topical tetracycline availability in the country, fusidic acid ointment (Forudine®, Dechra, Uldum, Denmark) was applied to the skin underneath the dressings every other day, because the perionyxis was painful and handling had to be limited. Crusted granulation tissue was apparent on the caudal side of the distal limbs but no oozing was noted (Supplementary Figure 3B). Dressings were changed every 2 days by the veterinarian and the owner. The dog had gained weight (9.4 kg) and was in good general health. A moisturizing cream (Trixera+®, Avène, Paris, France) was applied every day to facilitate skin healing. Oral doxycycline was maintained.

**Figure 3:**
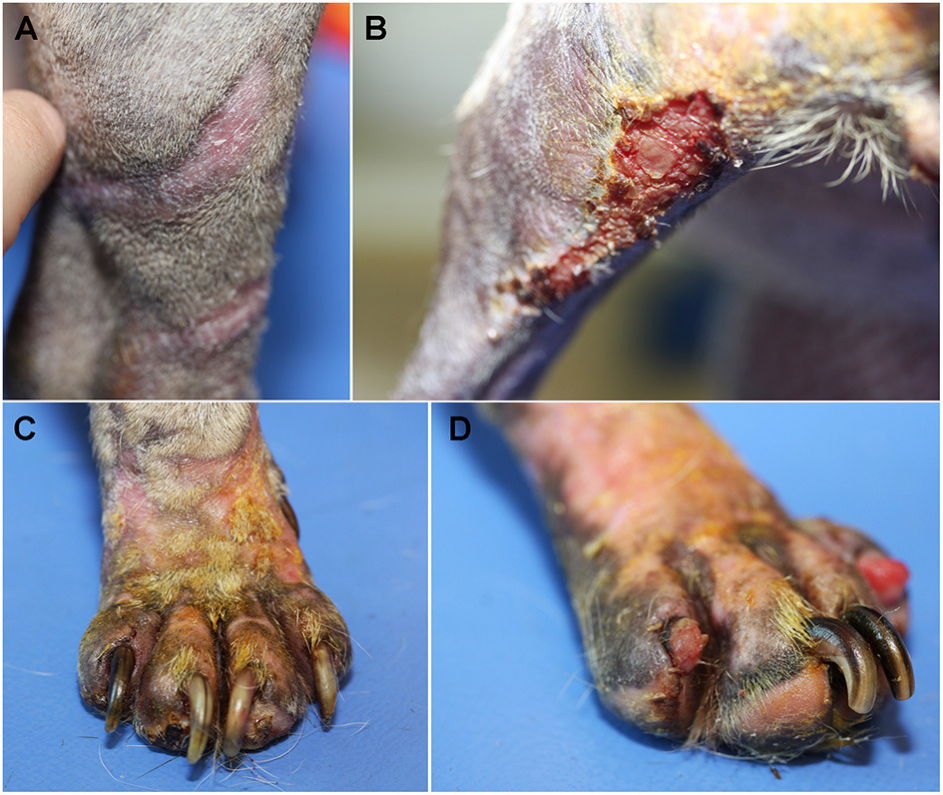
Clinical examination after 3 weeks. Healing was complete along the distal limbs **(A)**, crusty inflammatory lesions were observed on the back of the left thigh **(B)** and the extremities, particularly around the claws **(C)**. Onychomadesis was observed on digits II and IV of the left hind foot **(D)**.

After 3 weeks from starting doxycycline, the improvement was excellent, the distal limbs had completely healed (Figure 3A) but some crusty and inflammatory lesions were observed on the back of the thigh (Figure 3B) and the extremities, particularly around the claws (Figure 3C). Onychomadesis was observed on digits II and IV of the left hind foot (Figure 3D). Cytological examination of impression smears from crusty lesions on the caudal part of the hind legs revealed many neutrophils and rare bacteria. Doxycycline was maintained. Antiseptic shampoos (Douxo Pyo®, CEVA, Libourne, France) were prescribed twice weekly. Moisturizing cream was still applied once daily.

After seven weeks, the dog's weight was 9.8 kg, the skin had completely healed and noticeable hair regrowth had occurred (Supplementary Figure 4). Doxycycline was discontinued and antiseptic shampoos were continued once a week.

On the follow-up examination, 2 months after the doxycycline discontinuation, the dog was in a good body condition, and hair regrowth was almost complete.

## Discussion

TEN, also called ≪Lyell syndrome≫ is a rare life-threatening disorder reported in people and animals (1, 2). The hallmark of this syndrome is acute onset of epidermal detachment over a significant surface with associated systemic signs. It must be treated as an emergency. The prognosis is extremely poor, due mainly to consecutive sepsis. In humans, there is a high mortality rate of 30% and considerable long-term morbidity (16). The mortality rate for TEN in animals, based on relatively few case reports, is very high (7).

Antibiotic drugs, especially sulfonamides, penicillins, and cephalosporins are most often incriminated (4, 10, 17, 18). The pathomechanism is presumed to be immune-mediated, involving a cytotoxic CD8+ T-cell and natural killer (NK) cell activation against altered keratinocytes (7, 19). Soluble mediators including Fas-ligand, tumor necrosis factor-α (TNF-α), perforin, granzym, and mainly granulysin lead to confluent cell death (1, 20).

SJS and TEN are related conditions, distinguished by the percentage of BSA affected: <10% of the BSA affected is indicative of SJS, 10–30% indicates an overlap between SJS and TEN, and more than 30% of the BSA affected suggests TEN (21).

TEN is characterized by an acute onset of multifocal to generalized purpuric macules or patches, frequently extending to the mucocutaneous regions (2, 7). Some lesions rapidly progress to vesicular/bullous, necrotic and ulcerative lesions with detachable epidermis, and a positive pseudo-Nikolsky sign (21, 22). Footpads and digits are often involved (7). Systemic signs of illness (lethargy, depression, anorexia, and fever), severe pain and pruritus are common.

In this case, focal lesions of depigmentation were present on the upper lip and the perianal region was largely ulcerated. However, the oral cavity, which is a common localization (7), was not involved. Extension was very rapid with more than 30% of the BSA initially affected.

Differential diagnosis should include auto-immune subepidermal blistering diseases, accidental burns erythema multiforme, staphylococcal toxic shock syndrome and bacterial or viral infections (2, 7).

The animal's history and the physical examination are mandatory steps in the diagnosis. Other steps include questioning the owner about previous cutaneous reactions, any current treatments: antibiotics, nutraceuticals, deworming tablets, non-steroidal anti-inflammatory drugs, and any topical drug the dog could receive at the time of the reaction. Any previous exposure to the drug(s) suspected has to be investigated. Finally, the owner has to be questioned about the time between treatments and lesion development. When a drug reaction is suspected, any current xenobiotic has to be withdrawn immediately unless it represents an acutely life-threatening risk.

The Naranjo Scale was developed in human medicine to help standardize assessment of causality for all adverse drug reactions and establishes a causal association between a drug and an adverse event. It consists of 10 questions that are answered as either Yes, No, or “Do not know.” Different point values (−1, 0, +1, or +2) are assigned to each answer (8). Total scores range from −4 to +13; the reaction is considered definite if the score is 9 or higher, probable if 5–8, possible if 1–4, and doubtful if 0 or less. Other scales are available (16, 23). No equivalent exists for the dog, but their extrapolation is helpful.

For differential diagnostic purpose and suspecting TEN consequences, thoracic radiographs and abdominal ultrasound scans were obtained to rule out other organ damage secondary to possible sepsis or staphylococcal toxic shock syndrome (7, 24).

Interface dermatitis with extensive full-thickness epidermal necrosis and minor dermal inflammation are characteristic histological features although not pathognomonic for SJS/TEN (2, 3, 7, 19). However, in some occasions, this feature is not observed but rather a cytotoxic dermatitis with apoptosis of individual keratinocytes and satellitosis in multiples layers of the epidermis, signs that overlap histologically with erythema multiforme (EM) (2). In these cases, the clinician plays a key role in distinguishing EM from SJS/TEN. Multiple skin biopsies are recommended to confirm the diagnosis as it was shown that up to 25% of all skin biopsies lacked epithelium and thus were not diagnostic (2). Progressive lesions, ulcer margins, or the adjacent intact epidermis are sampling sites of interest (2, 3).

In our case, the dog arrived in a state of shock, dehydrated, and in extreme pain. Our first steps were to manage the pain, replace fluids and electrolytes, and prevent sepsis. As a drug reaction caused by amoxicillin was suspected, penicillins were withdrawn and replaced by antibiotics that have been less commonly associated with adverse cutaneous drug reactions (Supplementary Table 1). Constant patient monitoring, skin care and pain management were maintained for 15 days. Response to treatment was prompt and the dog improved the very next day. This improvement suggested that betalactams could be the initial trigger of the cutaneous reaction; furthermore, despite the use of marbofloxacin (resistant bacterial profile), the dog's condition improved, emphasizing the importance of the intensive care in the management.

According to the literature, adjunctive treatments have been used to counteract the immune mechanism behind SJS and TEN but in human medicine no survival benefits have been proved for glucocorticoids or high doses of human intravenous immunoglobulin (IV Ig) (1, 25). Ciclosporin A (CsA) has been associated with increased survival benefits in humans in many case reports and uncontrolled small studies (26, 27). Its effects may be attributed to its ability to inhibit cytotoxic T cells and its action on the apoptotic pathway which precludes the release of cytotoxic mediators (28). However, one study showed no significant difference in survival among all three treatment options of the study (CsA, IV Ig, supportive care) (29). A double-blinded controlled study is now required in veterinary medicine to determine the ideal dose and duration of treatment (6). In veterinary medicine, the cost of such treatment could be rapidly a brake, in particular when treating large breeds. Currently, the ideal immunomodulatory drug for the treatment of SJS/TEN remains elusive (6, 7, 30).

It is difficult to identify a candidate drug because provocation testing is not ethically acceptable, and because there is no fully validated *in vitro/ex vivo/ in vivo* models of drug allergy. In the present case, the use of the algorithms of drug causality for epidermal necrolysis (ALDEN) (23) and Naranjo scale (8) gave a score of 5 and 4 for amoxicillin, respectively, which corresponds to a probable causality. These results should be interpreted with caution as these algorithms have been developed for humans and have not been validated in dogs. Early withdrawal of all the xenobiotics the animal is exposed to at the time (only exception: acute life-threatening risk associated with the abrupt withdrawal molecule), referral to a specialized center, and optimal supportive treatment are crucial to the successful management of patients (2, 5, 7). In humans, identifying the causative drug can reduce the chance of death in TEN patients by 30% per day (7). TEN usually has a poor prognosis; the mortality rate in humans is 25–35% despite early diagnosis and relative easy access to intensive care units or burn units (1).

## Conclusion

In the present case, the good improvement of the dog's condition and the cutaneous lesions suggest that betalactams were the culprit drugs. As fluoroquinolones have never been reported to produce cutaneous drug reactions in dogs and cats, marbofloxacin was administered to prevent sepsis. A methicillin-resistant *Staphylococcus pseudintermedius* sensitive to fusidic acid, tetracyclines, chloramphenicol and resistant to marbofloxacin was isolated from the bacterial cultures. Doxycycline was prescribed, wound management using fusidic acid ointment and antiseptic treatments were maintained. The observed skin improvement and good general condition, despite the use of marbofloxacin was probably due to the intensive care and pain management. The present case illustrates the importance of rapid identification of any acute onset of necrolysis and epidermal detachment in animals. Drug treatments are most often involved and should automatically be stopped.

## Data Availability Statement

The original contributions presented in the study are included in the article/Supplementary Material, further inquiries can be directed to the corresponding author.

## Ethics Statement

Ethical review and approval were not required as no original research data was generated in this case report. All diagnostic and therapeutic procedures were performed by licensed veterinarians in the course of routine veterinary health management. Written informed consent was obtained from the owners for the participation of their animals in this study.

## Author Contributions

L-AL: investigation, follow-up, original draft, and review and editing. DC, EC-C, and M-CC: investigation and review and editing. MD: investigation (histopathology) and review and editing. CP: investigation, follow-up, and review and editing. All authors contributed to the article and approved the submitted version.

## Conflict of Interest

The authors declare that the research was conducted in the absence of any commercial or financial relationships that could be construed as a potential conflict of interest.

## Publisher's Note

All claims expressed in this article are solely those of the authors and do not necessarily represent those of their affiliated organizations, or those of the publisher, the editors and the reviewers. Any product that may be evaluated in this article, or claim that may be made by its manufacturer, is not guaranteed or endorsed by the publisher.
