# Supplementary material for: Case Report: Positive Outcome of a Suspected Drug-Associated (Immune Mediated) Reaction in a 4-Year-Old Male French Bulldog
Source: Front Vet Sci. 2021 Aug 20;8:728901. doi: 10.3389/fvets.2021.728901 (PMC8417874; doi:10.3389/fvets.2021.728901)

**Supplementary Figure 1. Histopathological examination of biopsies taken from the right forelimb and back thigh.** Severe extensive multifocal epidermal necrolysis with multifocal epidermal detachment and necrotic material within flaccid vesicles (A) and extension of ulcerations to the hair follicles (B). Mild heterogenous perivascular dermatitis with lymphocytes, neutrophils and plasma cells (C) and multifocal septal panniculitis (D) [H&E staining, magnification ×100 (a, b, d) and × 400 (c), bars = 100 µm (A, B, D) and 50 µm (C)].


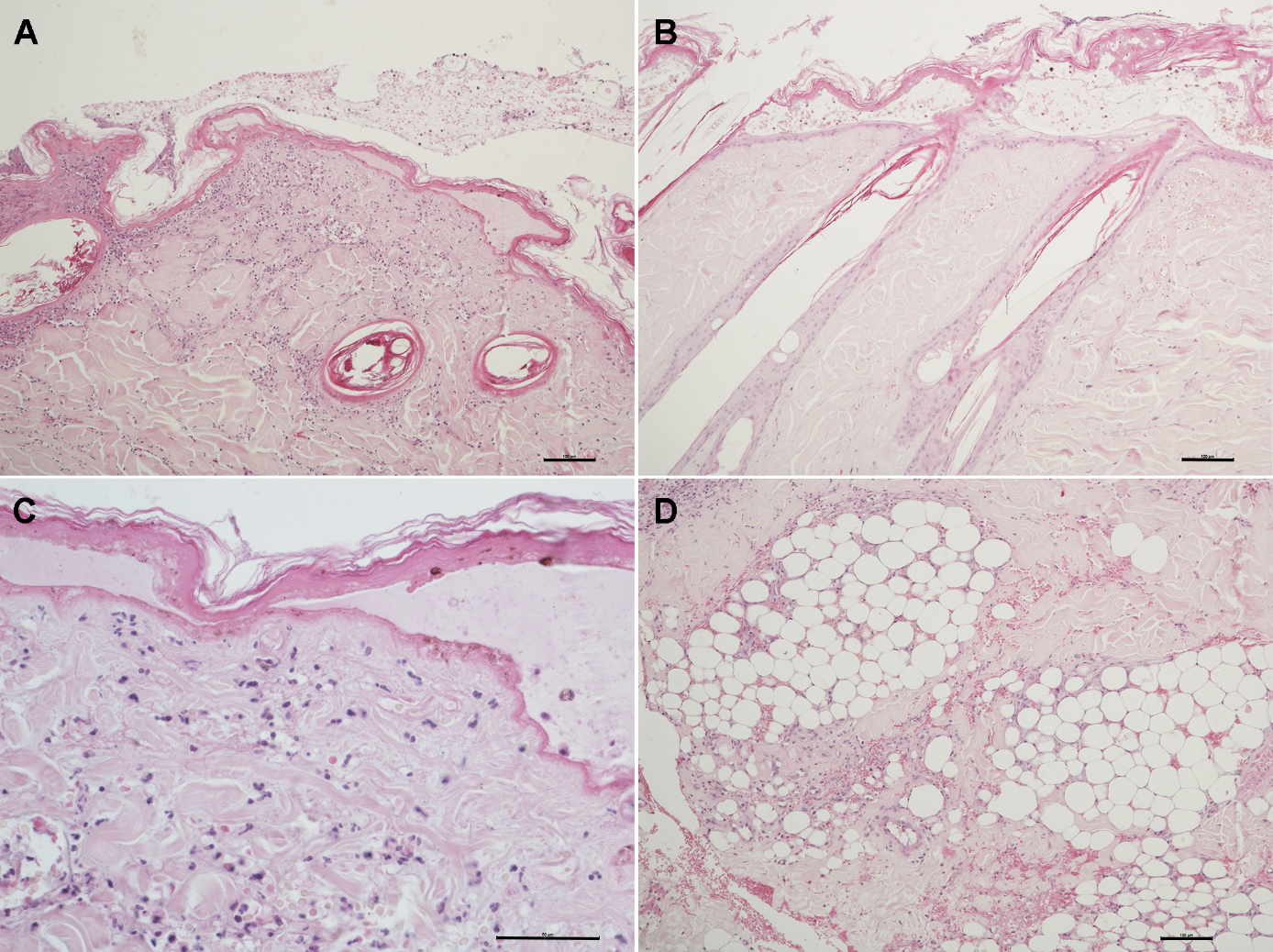

Supplement: Supplementary file 1 [file Data_Sheet_1.DOCX]
