# Supplementary material for: Case Report: Positive Outcome of a Suspected Drug-Associated (Immune Mediated) Reaction in a 4-Year-Old Male French Bulldog
Source: Front Vet Sci. 2021 Aug 20;8:728901. doi: 10.3389/fvets.2021.728901 (PMC8417874; doi:10.3389/fvets.2021.728901)

**Supplementary Figure 2. Clinical examination after two days.** Progression after gentle shaving, removal of detached epidermis and crusts, topical antiseptics and soft dressings (A). Granulation tissue was noticed (B).


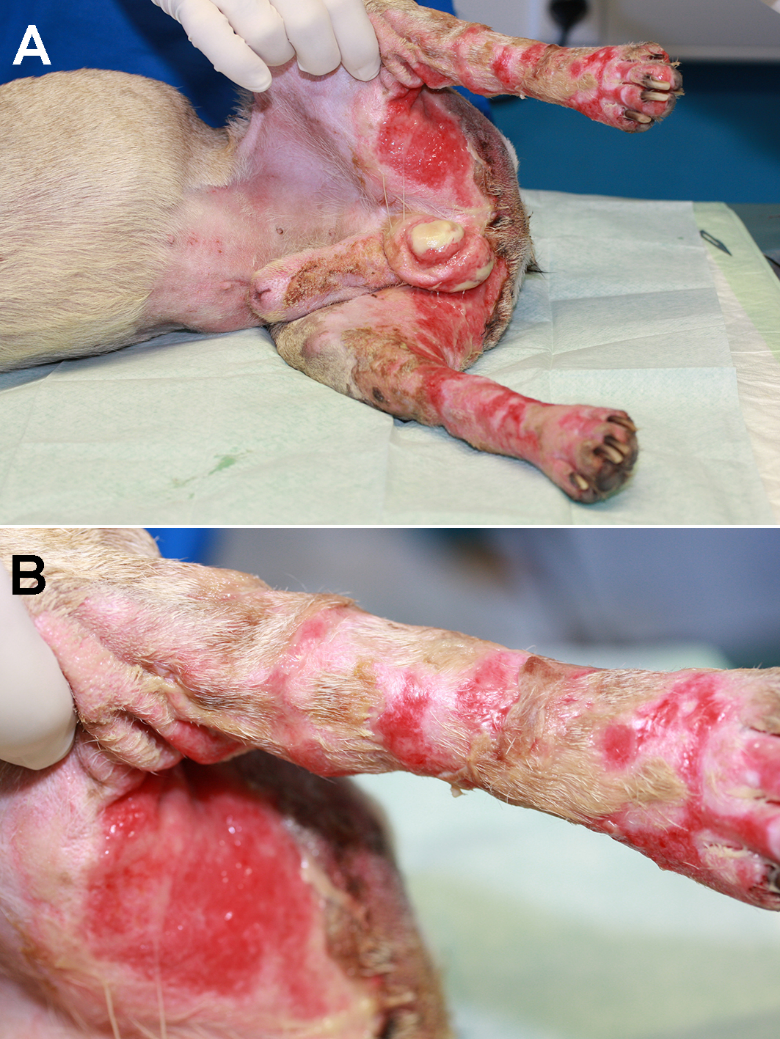

Supplement: Supplementary file 2 [file Data_Sheet_2.DOCX]
