# Supplementary material for: Case Report: Positive Outcome of a Suspected Drug-Associated (Immune Mediated) Reaction in a 4-Year-Old Male French Bulldog
Source: Front Vet Sci. 2021 Aug 20;8:728901. doi: 10.3389/fvets.2021.728901 (PMC8417874; doi:10.3389/fvets.2021.728901)

**Supplementary Figure 3. Clinical examination after 15 days.** Crusty perionyxis (A). Progressive healing of the hind limbs (B).


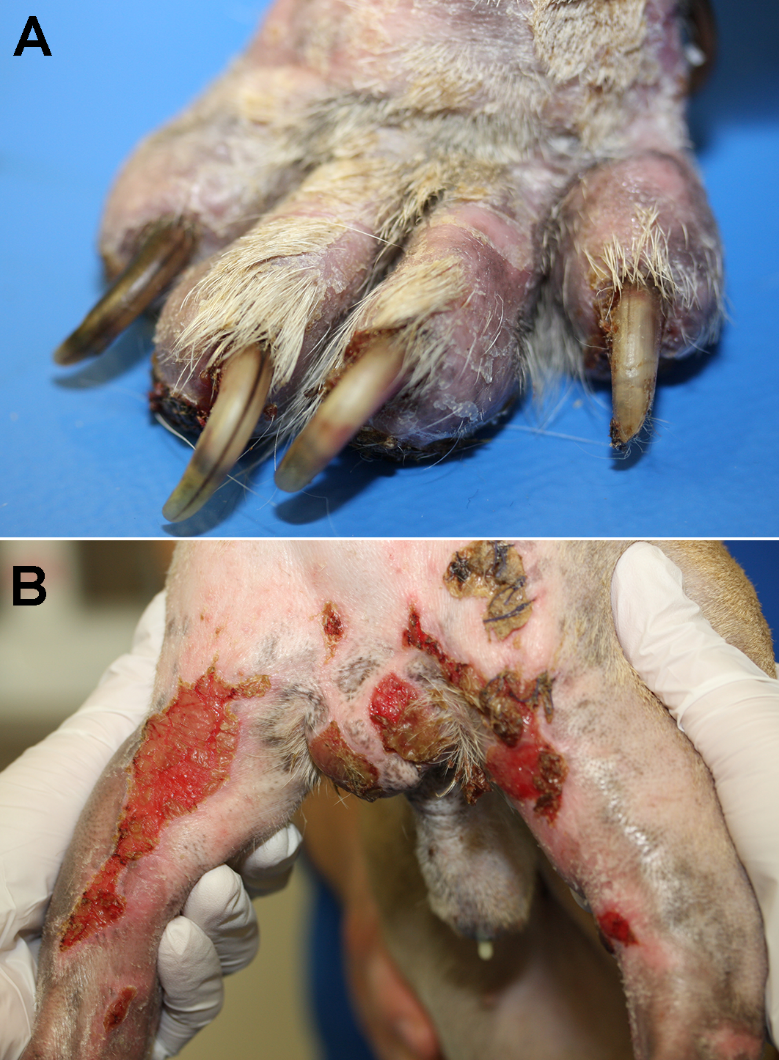

Supplement: Supplementary file 3 [file Data_Sheet_3.DOCX]
