# Supplementary material for: Case Report: Positive Outcome of a Suspected Drug-Associated (Immune Mediated) Reaction in a 4-Year-Old Male French Bulldog
Source: Front Vet Sci. 2021 Aug 20;8:728901. doi: 10.3389/fvets.2021.728901 (PMC8417874; doi:10.3389/fvets.2021.728901)

**Supplementary Figure 4. Clinical examination after seven weeks.** Complete healing with scar tissue and hair regrowth were observed on the caudal aspect of the thighs and perineum (A) and on all aspects of the limbs (B, C).


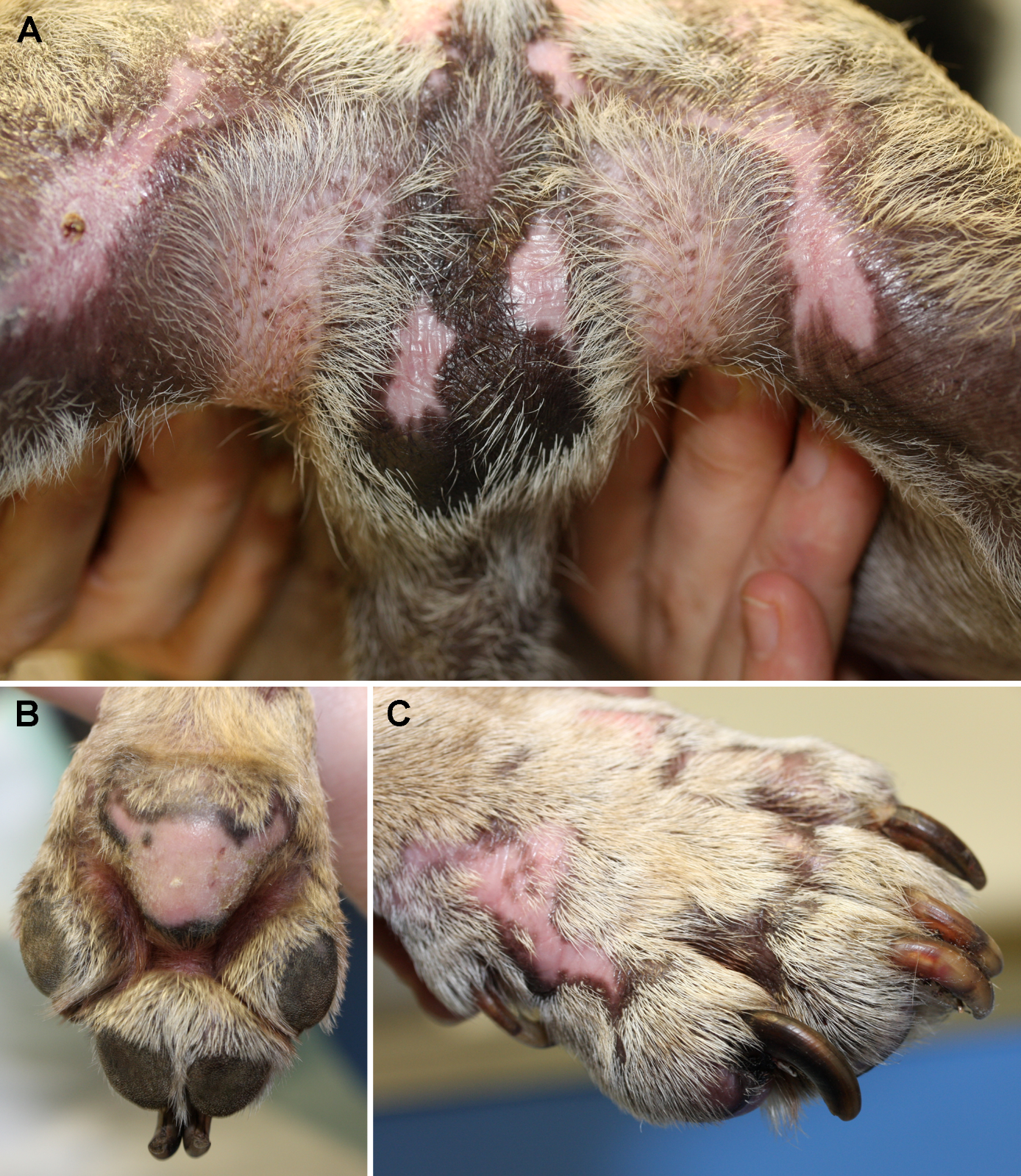

Supplement: Supplementary file 4 [file Data_Sheet_4.DOCX]
