# Supplementary material for: Case Report: Positive Outcome of a Suspected Drug-Associated (Immune Mediated) Reaction in a 4-Year-Old Male French Bulldog
Source: Front Vet Sci. 2021 Aug 20;8:728901. doi: 10.3389/fvets.2021.728901 (PMC8417874; doi:10.3389/fvets.2021.728901)
Supplement: Supplementary file 5 [file Table_1.DOCX]

**Supplementary Table 1.** Antibiotics reported to cause adverse cutaneous reactions in the dog.

| **Antibiotics frequently associated with adverse drug reactions** (1-3) | **Antibiotics rarely associated with adverse drug reactions** (2, 4) |
| --- | --- |
| Sulfonamides  Penicillins  Cephalosporins | Enrofloxacin  Erythromycin  Gentamicin  Lincomycin  Tetracyclin |

**References**

1. Medleau L, Shanley KJ, Rakich PM, Goldschmidt MH. Trimethoprim-sulfonamide-associated drug eruptions in dogs. *JAAHA* (1990) 26(3):305-11.

2. Miller Jr. WH, Griffin CE, Campbell KL. Toxic epidermal necrosis. In: Mosby E, editor. *Muller and Kirk's Small Animal Dermatology*. 7th ed. St Louis: Saunders (2012). p. 477-9.

3. Noli C, Koeman JP, Willemse T. A retrospective evaluation of adverse reactions to trimethoprim-sulphonamide combinations in dogs and cats. *Vet Q* (1995) 17(4):123-8. doi: 10.1080/01652176.1995.9694550. PubMed PMID: 8751272.

4. Saberi M, Kheirandish R, Shojaeepour S. Toxic epidermal necrolysis in a dog given enrofloxacin. *OJVR* (2014) 18(7):521-27.
